# The impact of different lenses on visual and musculoskeletal complaints in VDU workers with work-related neck complaints: a randomized controlled trial

**DOI:** 10.1186/s12199-017-0611-1

**Published:** 2017-03-16

**Authors:** Barbara Cagnie, Kayleigh De Meulemeester, Lieselotte Saeys, Lieven Danneels, Liesbet Vandenbulcke, Birgit Castelein

**Affiliations:** Department of Rehabilitation Sciences and Physiotherapy, Ghent University, De Pintelaan 185 3B3, 9000 Ghent, Belgium

**Keywords:** Lenses, VDU, Musculoskeletal complaints, Visual fatigue, Neck pain

## Abstract

**Objectives:**

The impact of wearing lenses on visual and musculoskeletal complaints in VDU workers is currently unknown. The goal of this study was 1) to evaluate the impact of wearing VDU lenses on visual fatigue and self-reported neck pain and disability, compared to progressive lenses, and 2) to measure the effect of both lenses on head inclination and pressure pain thresholds during the performance of a VDU task.

**Methods:**

Thirty-five eligible subjects were randomly assigned to wear progressive VDU lenses (VDU group) (*n* = 18) or progressive lenses (P group) (*n* = 17). They were enquired about visual complaints (VFQ), self-perceived pain (NRS) and disability (NDI) at baseline (with old lenses), and 1 week, 3 months and 6 months after wearing their new lenses. In addition, Forward Head Angle (FHA) and PPTs were assessed during and after a VDU task before and 6 months after wearing the new lenses. A short questionnaire concerning the satisfaction about the study lenses was completed at the end of the study.

**Results:**

In both groups, visual fatigue and neck pain was decreased at 3 and 6 months follow up, compared to baseline. All PPTs were higher during the second VDU task, independent of the type of lenses. The VDU group reported a significantly higher suitability of the lenses for VDU work.

**Conclusion:**

It can be concluded that there is little difference in effect of the different lenses on visual and musculoskeletal comfort. Lenses should be adjusted to the task-specific needs and habits of the participant.

## Introduction

Over the past years, the use of computers and other digital electronic devices has increased dramatically. The use of these visual display units (VDU) is often accompanied by physical complaints of the neck, shoulder, forearm and hand, with incidence and prevalence rates ranging from 21 to 36% [1–3].

Many VDU workers not only experience musculoskeletal complaints, but also eye discomfort and vision problems when working with the computer for a long period [4]. This phenomenon is called Computer Vision Syndrome (CVS) and may be caused by poor lighting, glare on a digital screen, poor seating posture, uncorrected vision problems, improper viewing distances, or a combination of these factors.

Few studies have investigated the impact of inappropriate vision on neck pain in VDU workers. Myopia, or nearsightedness, is a condition in which objects on a far distance appear blurred. This condition generally starts during childhood and progresses until the age of 20. Hyperopia (farsightedness) and astigmatism are two other conditions that frequently contribute to the development of visual symptoms when using a computer or digital screen device. All these conditions can co-occur with presbyopia, the normal age-related loss of near focusing ability.

Today, a large variety of lenses exists to correct several kinds of vision problems. Single-vision lenses are prescribed if one of the above mentioned conditions occur solitarily. When two or more conditions are present at the same time, bifocals or progressive lenses can be prescribed. However, most of these lenses are not suitable for computer work as their intermediate zone is not large enough to enable VDU work comfortable. As these lenses are not perfect for computer work, VDU workers might compensate their blurred vision by leaning forward, or by tipping their head to look through the bottom portion of their lenses, which can result in neck complaints.

With the increase of computer use, lenses designed specifically for computer use have been developed. These lenses provide a clear vision of the intermediate zone at a distance of approximately 70 cm, which allows a clear view of the computer screen. Table 1 gives a clear overview of the main differences between progressive lenses and office lenses.

**Table 1:** Overview of the difference in function between progressive lenses and office lenses

	Progressive lens	ZEISS Officelens
Depth of field	Unlimited depth of field, clear vision at every viewing distance	Clear vision at near and intermediate viewing distance
Intermediate and near zone	Small intermediate and near zone to offer large distance zone for uninterrupted far vision and dynamic vision	Large near and intermediate zones
Gaze stabilisation	Gaze stabilization may take longer due to difficulties finding the areas of optimal focus	Superior balance of optics because lens design has a larger available lens area - no distance zone necessary
Viewing distance	Unlimited maximum viewing distance > 6 m	Limited viewing distance to 1 m, 2 m or 4 m
Addition power	Full change in addition power needed	No full change in addition power needed

Only a few studies have evaluated the impact of wearing single- or progressive lenses on body posture, muscle overload and pain during VDU work [5–9]. Becker et al. [5] compared progressive lenses with single-vision lenses and found that wearing progressive lenses leads to greater degrees of change in forward head posture and occipital extension. In an observational study of Jaschinski et al. [7], a significantly larger head inclination was found while wearing progressive lenses, compared to wearing single-vision lenses for far or near vision. In another study of Jaschinski et al. [6], a significantly lower head inclination and better vision of the computer screen was found while wearing VDU lenses, compared to progressive lenses. Horgen et al. [9] compared 3 types of progressive VDU lenses and single-vision lenses during a VDU task and found no significant differences in muscle load between the different lenses by using electromyography. Later, Horgen et al. [8] set up a randomized controlled trial (RCT) and compared the effect of wearing different lenses for 1 year: 3 types of progressive VDU lenses and single-vision lenses were compared. A significant reduction of neck pain was found with one type of the VDU lenses as well as high rates of satisfaction with two types of the VDU lenses and the single-vision lenses. However, the above mentioned studies have some limitations, including limited follow-up and lack of more objective measurements. In order to fulfill the need for more objective measurements and larger follow-up periods, an RCT was set up with the following research questions.

1. What is the impact of wearing progressive VDU lenses, compared to progressive lenses, on self-reported neck pain and disability, and visual fatigue?
2. What is the impact of wearing progressive VDU lenses compared to progressive lenses, during a VDU task, on head inclination and pressure pain thresholds?

## Methods

### Subjects

Male and female VDU workers working in a financial holding in Ghent were recruited for this study. The supervising physician of the study screened all subjects for eligibility to participate in the study. The subjects were included if they met the following criteria: (1) aged between 45 and 65 years, (2) performing VDU work for at least 4 h a day and 20 h a week (3) experiencing work-related neck/shoulder pain or discomfort of more than 30 days during the last year and (4) having a difference in spectacle correction for presbyopia and myopia of minimum 1.5 dioptres. Subjects were excluded in case of (1) wearing VDU lenses before the study, (2) having active eye diseases that could not be corrected with eyelenses and (3) using medication that strongly influences eye or muscle function.

All subjects signed an informed consent and the study was approved by the Local Ethics Committee of Ghent University Hospital.

### General design

The study was conducted from April 2015 till December 2015. Eligible subjects were asked to fill out a questionnaire concerning visual complaints (visual fatigue questionnaire - VFQ) and self-perceived pain (numeric rating scale - NRS) and disability (neck disability index - NDI). First, subjects were tested with their current (old) lenses in a landscape office while performing a **20-minute VDU task** (task 1). A **2D video analysis** was performed during the task to measure the Forward Head Angle (FHA) [10]. The **PPT** of the trapezius, levator scapulae and infraspinatus muscles was measured after the VDU task. After these first investigations, subjects were randomly assigned to the treatment or control group using a block randomization in order to obtain an equal size and sex distribution in each group. The treatment group was given progressive VDU lenses (ZEISS Officelens Plus), specially designed for VDU work, whereas the control group was given progressive lenses (ZEISS Precision Plus). Spectacle frames of Silhouette® were used and an eye examination was performed by an experienced optician optometrist.

**Fig. 1:**
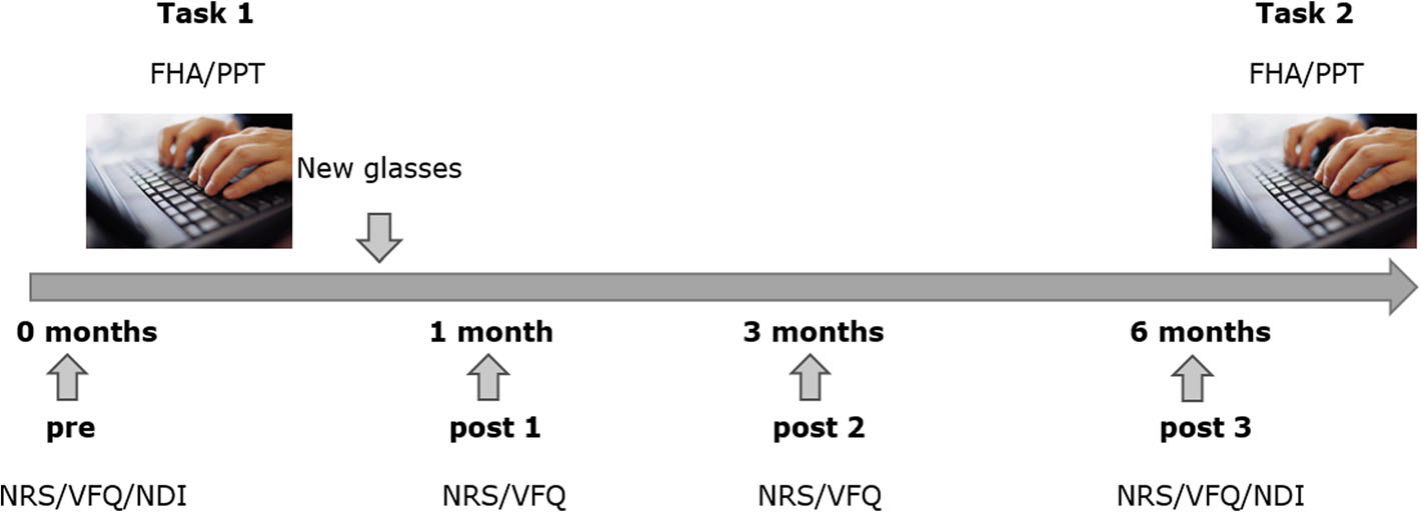
Overview of the general study procedure

Within 1 month after the first test moment, the participants received their new lenses and were asked to wear these for 6 months during VDU work. The VFQ and NRS were inquired 1 week (post1), 3 (post2) and 6 (post3) months after wearing their new lenses. Self-perceived disability was enquired after 6 months wearing the new lenses (post3). At 6 months follow up, the test procedure was repeated while wearing the new lenses (task 2). A short questionnaire concerning the satisfaction about the study lenses was completed at the end of the study. An overview of the study procedure is given in Fig. 1.

### VDU task

All participants were tested in the morning and were asked not to use alcohol, nicotine and caffeine 24 h and painkillers 48 h before this VDU task. They were also asked not to perform intensive physical activity 24 h before the task. Participants were tested with the line of sight parallel to the window. Glare was reduced by the use of sun blinds and sufficient light was provided. Computer screen, chair and table height were adjusted individually with the top of the screen at or just below eye level and 90° of flexion in the elbows, hips and knees. The subjects performed a typing task during 20 min. This task consisted of copying a predefined, unknown text that was given on the left side of the screen. The subjects were asked to type this text in a blank document at the right side of the screen, using the computer mouse only to scroll down in both documents.

### Outcome measures

#### Primary outcome measures

##### Self-reported visual fatigue

The Visual Fatigue Questionnaire (VFQ) was enquired at four different time points (pre-post1-post2-post3). This questionnaire consists of 15 questions, which have to be answered on a VAS ranging from zero to ten [11]. It enquires eye strain, impaired vision, impaired eye surface and problems apart from the eyes, e.g. headache. The content validity index is 0.75 and the correlation between the results of the questionnaire and the Visual Fatigue Meter, a device that measures the eye fatigue changes, is −0.87 [11]. Scores below 0.65 indicate ‘no fatigue’ , between 0.66 and 2.36 ‘low fatigue’ , between 2.37 and 3.88 ‘moderate fatigue’ and scores above 3.89 ‘severe fatigue’.

##### Self-perceived pain

General neck pain was assessed on a 11-point Numeric Rating Scale (NRS) (0, no pain; 10, worst possible pain) at four different time points (pre-post1-post2-post3). At these time points, subjects were asked to rate the pain that they experienced in the preceding week.

##### Self-reported disability

The Neck Disability Index (NDI) was completed at the beginning (pre) and at the end (post3) of the study [12]. The NDI consists of ten questions concerning pain and daily living activities such as lifting, reading and driving, and each item is scored from 0 to 5. The maximum score is therefore 50. A score between 5 and 14 represents a mild disability whereas a score between 15 and 24 is interpreted as a moderate disability. NDI scores higher than 25 reflect a severe disability.

#### Secondary outcome measures

##### Head posture

A 2D video analysis of the head posture was performed during the task using a Logitech® webcam with a ZEISS® Tessar HD 1080p lens and MaxTRAQ and MaxMATE software. FHA was measured by placing markers on C7 and the tragus and calculating the angle between the vertical and the line connecting the tragus and the C7 marker [10]. The webcam was positioned on a tripod, perfectly horizontal with a level meter and at a standardized distance from the participant. The participant was recorded for 15 s during the 5^th^, 10^th^ and 15^th^ minute of the VDU task (45 s in total). The mean of the three measurements was used for further analysis.

##### Pressure pain threshold

The Wagner™ FDX 50 hand-held pressure algometer is a digital device that measures the level at which pressure (N) becomes unpleasant, also known as the Pressure Pain Threshold (PPT). The subject had to sit down relaxed with the hands on the thighs. The investigator applied an increasing pressure on a predetermined point of the muscle at a rate of approximately 1 N/s with a 1 cm^2^ round rubber tip. The subject was instructed to say “yes” when the comfortable pressure changed into an unpleasant pressure. The algometer was applied on the trapezius muscle (the middle between C7 and the posterior part of the acromion), the levator scapulae muscle (superior and medial of the superior angle of the scapula) and the infraspinatus muscle (the angle between the spina scapulae and the lateral margin of the scapula), each time on both sides of the body. Measurements were performed twice after the VDU task.

Walton et al. [13] demonstrated substantial to near perfect reliability (ICC = 0.76 –0.97) of the PPT of the trapezius in people with and without acute neck pain. In the current study all measurements were performed by the same rater.

##### Satisfaction

A short questionnaire concerning the satisfaction about the study lenses was completed at the end of the study. First, the overall satisfaction was questioned. Second, participants had to indicate to which extent the study lenses were appropriate to use for VDU work, for near (e.g. reading) and far vision (e.g. looking at colleagues in the same working area). A score had to be given on a five-point likert scale ranging from ‘not at all’ to ‘extremely satisfied’.

### Statistical analysis

Data were analysed using IBM® SPSS® Statistics version 23.0. An Independent Samples *T* Test was applied to reveal significant group differences and to assess satisfaction scores.

In order to investigate the impact of wearing progressive VDU lenses, compared to progressive lenses on self-reported visual fatigue, neck pain and disability, analyses of variance with repeated measures was applied. For all outcome measures, the between factor was *group* with two levels (VDU lenses and progressive lenses) and the within factor was *time* with four levels (pre, post1, post2 and post3), except for the NDI (pre and post3).

In order to evaluate differences in head inclination and PPTs during a VDU task between wearing progressive VDU lenses and progressive lenses, a similar analysis of covariance with repeated measures was performed. For all outcome parameters, the between factor was *group* (VDU lenses vs. progressive lenses), and the within factor was *time* (task 1 and task 2). A post-hoc pairwise comparison was performed using a Bonferroni correction. An alpha level of 0.05 was applied to all the data in determining significant differences.

## Results

### Participants

Sixty-five participants were screened for eligibility in February and March 2015 and 35 participants (14 females and 21 males) met the eligibility criteria. Eligible participants were randomized into the VDU lenses group (*n* = 17; mean age ± SD, 51.09 ± 4.19 years) or the progressive lenses group (*n* = 18; mean age ± SD, 53.67 ± 3.97 years). No significant differences in demographic characteristics were found between both groups. Seven participants dropped out during the course of the study, resulting in a total of 15 subjects in de VDU lenses group and 13 subjects in the progressive lenses group, who completed the study. The two dropouts from the VDU lenses group noted that the lenses were appropriate to see the computer screen clearly, but one could not see his colleagues clearly and the other could not see his second computer screen appropriately. All five dropouts from the progressive lenses group stated that the lenses were not suitable for VDU work.

#### Primary outcome measures

Mean ± SD of all primary outcomes measures are described in Table 2. No significant time x group interaction effects were found for all primary outcome measures (*P* > 0,05).

**Table 2:** Descriptive statistics of VFQ, NRS and NDI of the VDU and P group at different time points

Outcome measure	Group	Pre	Post1	Post2	Post3
VFQ	VDU	2.30 ± 1.33	1.87 ± 1.11	1.03 ± 0.66	0.99 ± 1.08
	P	2.40 ± 2.03	2.58 ± 1.70	2.15 ± 1.64	1.81 ± 1.42
NRS	VDU	3.33 ± 1.50	2.93 ± 1.28	2.13 ± 1.06	1.87 ± 1.41
	P	3.15 ± 2.15	4.31 ± 2.02	3.69 ± 1.84	2.92 ± 1.61
NDI	VDU	9.69 ± 5.79			6.87 ± 6.98
	P	8.64 ± 4.76			7.58 ± 4.06

##### Self-reported visual fatigue

**Fig. 2:**
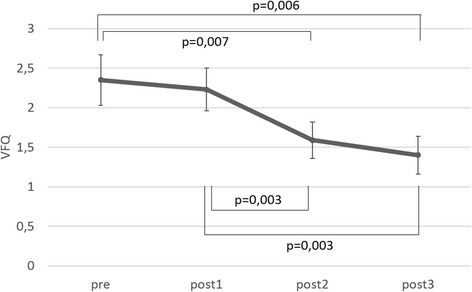
Mean ± SE based on estimated marginal means of the VFQ at the different time points

There was a main effect for time (*F* = 7.947; *p* = 0.001), but not for group (*F* = 2.362; *p* = 0.136). Post hoc analysis revealed no significant differences between pre and post 1 and between post2 and post3, but demonstrated significantly lower values in post2 and post3 compared to pre and post1 (Fig. 2).

##### Self-perceived neck pain

**Fig. 3:**
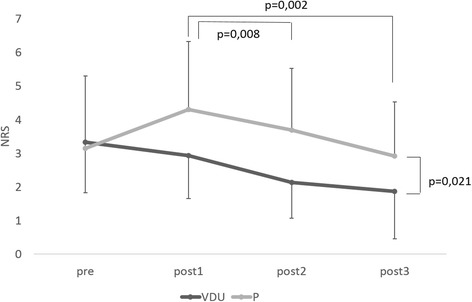
Mean ± SE based on estimated marginal means of the NRS at the different time points in the VDU and P group

There was a main effect for time (*F* = 3.561; *p* = 0.034), and for group (*F* = 6.031; *p* = 0.021). Post hoc analysis revealed significantly lower scores at post2 and post3 compared to post1, independent of the type of lenses and a significantly lower score in the VDU group, independent of time (Fig. 3).

**Fig. 4:**
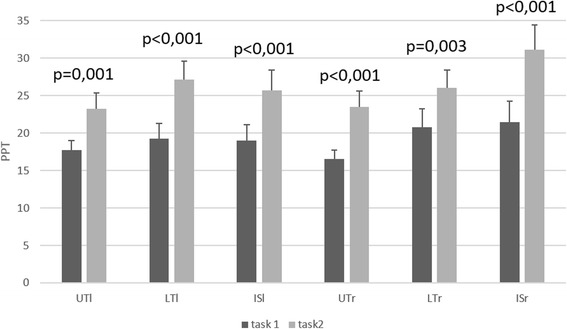
Mean ± SE based on estimated marginal means of the PPT of the left and right UT, LT and LS after task 1 and task 2

##### Self-reported disability

No significant main effects for time (*F* = 0.021; *p* = 0.887) and group (*F* = 0.193; *p* = 0.664) were identified for self-reported disability.

#### Secondary outcome measures

Mean ± SD of all secondary outcomes measures are described in Table 3. No significant time x group interaction effects were found for all secondary outcome measures (*P* > 0,05).

**Table 3:** Descriptive statistics (mean ± SD) of head posture and PPT of the VDU and P group at different time points

Outcome measure	Groups	Task 1	Task 2
Head posture			
	VDU	63.20 ± 11.00	62.15 ± 9.80
	P	57.62 ± 8.45	57.08 ± 7.94
Pain pressure threshold			
Trapezius left (N/s)	VDU	16.34 ± 7.25	19.95 ± 9.75
	P	19.07 ± 6.48	26.54 ± 12.42
Trapezius right (N/s)	VDU	15.83 ± 5.36	20.02 ± 6.95
	P	17.29 ± 7.11	26.99 ± 14.15
Levator scapulae left (N/s)	VDU	17.03 ± 10.31	24.22 ± 14.03
	P	21.44 ± 10.88	30.01 ± 12.04
Levator scapulae right (N/s)	VDU	19.30 ± 11.47	25.31 ± 10.44
	P	22.22 ± 15.08	26.75 ± 14.32
Infraspinatus left (N/s)	VDU	17.22 ± 10.44	22.15 ± 12.75
	P	20.83 ± 11.92	29.17 ± 15.91
Infraspinatus right (N/s)	VDU	20.49 ± 13.38	27.81 ± 14.03
	P	22.35 ± 16.45	34.49 ± 20.21

##### Head posture

No significant main effects for time (*F* = 0.921; *p* = 0.347) and group (*F* = 2.210; *p* = 0.150) were found for FHA.

##### Pain pressure threshold

There was a significant main effect for time for all PPTs (*p* < 0.004) (Fig. 4) but not for group (*p* > 0.05). All PPTs were significantly higher when using the new lenses (task 2), independent of the type of lenses.

##### Satisfaction

No significant difference in overall satisfaction between the two types of lenses was found (*p* = 0.072) (Table 4). The VDU lenses group reported a significantly higher suitability of the lenses for VDU work (*p* = 0.001) while the progressive lenses group reported a significantly higher suitability for far vision (*p* < 0.001). For the satisfaction for close vision no significant difference was found between the two types of lenses (*p* = 0.115).

**Table 4:** Descriptive statistics (mean ± SD) of the satisfaction questionnaire (five point scale) of the VDU and P group

	VDU	P	*p*-value
Overall satisfaction	4.22 ± 0.81	3.46 ± 1.45	0.072
Far vision	1.83 ± 1.04	4.54 ± 0.66	<0.001
Intermediate vision	4.67 ± 0.59	3.38 ± 1.26	0.001
Close vision	4.28 ± 0.75	3.69 ± 1.25	0.115

## Discussion

The aim of this RCT was to investigate the influence of wearing progressive VDU lenses, compared to progressive lenses on self-reported pain, disability and visual fatigue and to investigate the impact of these lenses on pain sensitivity and head inclination during a VDU task. It can be concluded that both lenses have a significant impact on different outcome measures but that there is little difference in visual and musculoskeletal comfort between wearing progressive and VDU lenses.

In both groups there was a significant decrease in self-reported **visual fatigue and neck pain** at 3 and 6 months follow up compared to baseline (for visual fatigue only) and 1 week after wearing the new lenses (for both outcome measures).

**Visual fatigue** has been assessed in previous studies using mainly one dimensional measurements, but none of these studies have used the VFQ, as this is a rather new measurement tool. Similar to the results of the general neck pain, the VFQ at 3 and 6 months follow up showed only small, but significant changes in both groups.

The **baseline NRS value** for neck **pain intensity** and the decrease in intensity is similar to a previous study of Horgen et al. [8] who followed VDU-workers during a 12-month period wearing different types of lenses. Despite this significant difference, the decrease in NRS was lower than the MCID of 1.5 points, which is required to obtain a small detectable patient-perceived change [14]. These small changes could amongst others be attributed to the fact that the general level of pain was relatively low at baseline.

Neither of the groups’ results showed significant changes in **self-reported neck disability**. All participants had a mild limitation (NDI scores between 5 and 14) at the start of the experiment which may explain why no significant changes in NDI scores were found. On the other hand, NDI scores indicating mild limitation are rather common in patients with work related neck pain.

There were no changes in **FHA** during the VDU tasks. Past research included only one study that compared VDU lenses with progressive lenses. Jaschinski et al. [6] found a significantly lower head inclination when wearing VDU lenses which is conflicting with the results of this study.

All **PPTs** were higher during the second VDU task, independent of the type of lenses. This increase in PPT could be attributed to the decrease in general neck pain and visual fatigue

The VDU group reported a significantly higher **suitability** of the lenses for VDU work while the P group reported a significantly higher suitability of the lenses for far vision. According to Jaschinski et al. [6], vision of the computer screen was judged significantly better with VDU lenses and far vision was judged better with progressive lenses which is comparable with the results of this study.

Besides the quantitative analyses, some subjects in the VDU lenses group reported that the lenses were not suitable for far vision and that social contact with colleagues in the same working area was strongly reduced. Others reported that the lenses were not suitable to walk with in the working area because of dizziness and reduced depth perception. In the VDU lenses group there was still a need to use progressive lenses for other tasks then VDU work. Some subjects reported that it was difficult to switch between both types of lenses during the day.

In the progressive lenses group several subjects reported that the lenses were not suitable for VDU work and that the computer screen had to be placed in another position or that the head had to be positioned in an unnatural posture.

The present results should be viewed within the limitations of the study. There was no blinding possible for the intervention because of the nature of the study. VDU lenses can only be used during VDU work, whereas progressive lenses can be used all day long, so the participants couldn’t be blinded.

Another limitation of this RCT was that the kind of lenses that participants used at the beginning of the study was not taken into account into the analysis. When entering the study, sixteen participants used progressive lenses, 13 used reading lenses, 2 used lenses for far vision and 4 didn’t use any lenses at all during VDU work. In the two groups a comparable number of subjects already used similar kinds of lenses at the beginning of the study.

In future studies, a more in depth qualitative analysis of comparing different lenses is needed. Bigger sample sizes, in order to meet a high dropout rate, is needed. In addition, the kind of lenses that are used at the beginning of the study should be taken into account as well in order to have a more homogeneous population.

It can be concluded that there is little difference in effect of the different lenses on visual and musculoskeletal comfort. Lenses should be adjusted to the task-specific needs and habits of the participant.
